# Supplementary material for: A Review of Keratoconus Cross-Linking Treatment Methods
Source: J Clin Med. 2025 Mar 3;14(5):1702. doi: 10.3390/jcm14051702 (PMC11899953; doi:10.3390/jcm14051702)
Supplement: Supplementary file 1 [file jcm-14-01702-s001.zip › jcm-3438002-supplementary.pdf]

First Author	Year	Study Design	Origin of study	Population	Epithelial removal	Main Findings	Funding Sources	Conflicts of Interest
Davidson et al.	2014	Rev	UK	adult	N/A	Etiology based on primary and secondary changes, i.e., inflammatory and degenerative effects. Gene loci suggested to contribute.	UNK	none
Santodomingo-Rubido et al.	2022	Rev	Japan, Spain, Australia	pediatric, adult	off, on	Mild cases are treated with spectacles, moderate with contact lenses, while severe cases may require corneal surgery.	UNK	UNK
Flockerzi et al.	2021	R	Germany	adult	N/A	Stage distributions in all age groups similar. Early KC rather becomes manifest in the posterior than the anterior corneal curvature, advanced stages of posterior corneal curvature coincide with early and advanced stages of anterior corneal curvature.	no external funding	Financially none. Travel grant / seminar invitation. Simultaneous membership of the Institute of Experimental Ophthalmology, Saarland University Medical Center (Homburg, Germany), now an employee of the Amiplant GmbH (Schnaittach, Germany).
Hwang et al.	2018	R	South Korea	adult	N/A	Age and sex-specific prevalence and incidence of keratoconus among South-Korean population. Incidences peak in the late 20s in males, and in the early 20s in females. Overall prevalence and incidence rates did not present a gender predisposition.	none	none
Gordon-Shaag	2015	Rev	Israel, Hong Kong,	pediatric, adult	N/A	Both environmental and genetic factors contribute to the pathogenesis of KC.	UNK	none
Angelo et al.	2022	Rev	New Zealand	pediatric, adult	off, on	Accelerated protocols as effective as standard with less cost burden. Combined protocols allowed for simultaneous addressing of disease progression and visual rehabilitation. CXL application has expanded showing benefit in treating infective keratitis and bullous keratopathy.	UNK	none
Wollensak et al.	2003	P	Germany	pediatric, adult	off	Dreseden protocol shown to be effective in stopping the progression of keratoconus.	UNK	UNK
Shalchi et al.	2015	Rev	UK, USA	pediatric, adult	off, on	Although TE CXL has fewer complications, it is less effective, particularly in stabilising or improving Kmax.	UNK	none
Raiskup et al.	2015	R	Germany, Czech Republic	pediatric, adult	off	Corneal CXL was effective in treating progressive keratoconus, achieving long-term stabilization of the condition.	no external funding	none
Poli et al.	2015	P	France	pediatric, adult	off	Stabilized or improved corneal ectasia, significant improvement in CDVA and long-term stability of keratometry, as compared to baseline preoperative values. Endothelial and retinal tolerance confirmed, long-term follow-up raises the question of a apossible increase in IOP.	none	none
O'Brart et al.	2015	P	UK	pediatric, adult	off	Improvements in topographic and wavefront parameters evident at 1 year were seen to continue to improve at 5 years and were maintained at 7 years. No treated eyes progressed over the 7-year follow-up period.	none	none
Eslami et al.	2022	R	Canada	pediatric, adult	off	Progressive improvement of refractive and keratometric indices. Steady decline of corneal aberrations with long-term follow-up, more pronounced in more severe patients.	no external funding	none
Iqbal et al.	2019	R	Egypt	pediatric, adult	off	Good effectiveness and stability, that halted KC progression along the 5-year follow-up. Unexpected improvement in the KC refractive states mainly the spherical and SE components.	UNK	none
Sachdev et al.	2015	CS	India	UNK	off	Intraoperative stromal augmentation with refractive lenticules obtained after small-incision lenticule extraction for myopia allowed safe and effective CXL in keratoconic eyes with ultrathin corneas.	no external funding	none
Gu et al.	2014	CT	China	adult	off	CXL with a hypoosmolar riboflavin solution seemed to be a promising method for thinner corneas. Longer follow-up and larger patient series needed.	UNK	none
Wittig-Silva et al.	2008	RCT	Australia	UNK	off, on	Early findings from this randomized controlled trial indicate that all treated eyes experience temporary stabilization following CXL.	UNK	none
Mark et al.	2014	CT	Germany, Cameroon	pediatric, adult	off	Significant, unexpected differences between these African eyes compared with European eyes, unexpected intraoperative swelling. Further studies are necessary.	Funding by the German Ophthalmological Society.	none
Gadelha et al.	2009	CT	Brazil	adult	off	Effective in controlling painful symptoms of bullous keratopathy. Maintenance of corneal thickness and visual acuity.	UNK	UNK
Caporossi et al.	2006	P	Italy	adult	off	Refractive reduction of about 2.5 D in mean spherical equivalent, topographically reduction of mean K. Surface aberrometric analysis improved in morphologic symmetry with significant reduction in comatic aberrations.	none	none
Chowdhury et al.	2020	RCT	UK	pediatric	off	Description of statistical aspects of KERALINK providing on the relative effectiveness comparing epithelium-off CXL with standard care.	Supported by the Efficacy and Mechanism Evaluation Programme (reference 14/23/18), an MRC and NIHR partnership.	none
Hersh et al.	2017	P, RCT	USA	pediatric, adult	off, on	In the CXL treatment group, the maximum keratometry value decreased by 1.6 D from baseline to 1 year, whereas keratoconus continued to progress in the control group. In the treatment group, the maximum keratometry value decreased by 2.0 D or more in 31.4% and increased by 2.0 D or more in 5.6%. Corneal haze was the most frequently reported CXL-related adverse finding.	Peschke Meditrade	Collaboration with Avedro, Inc (Waltham, MA, USA) and others
Hersh at al.	2011	P, RCT	USA	pediatric, adult	off, on	Collagen crosslinking was effective in improving UDVA, CDVA, the maximum K value, and the average K value. Keratoconus patients had more improvement in topographic measurements than patients with ectasia.	none	none
Legare et al.	2013	R	Canada	pediatric, adult	off	CXL is a safe and effective stabilizing strategy for progressive mild-to-moderate keratoconus with significant improvement of the UDVA. Trend toward improvement of BCVA in patients with lower preoperative values.	none	none
Khattak et al.	2015	P	Saudi Arabia, Ireland	pediatric, adult	off	CXL is an effective treatment in eyes with progressive Keratoconus by significantly reducing average mean K read- ings and decreasing asphericity, after one year follow-up.	UNK	none
Hashemi et al.	2013	P	Iran	pediatric, adult	off	Stabilized UCVA, refraction, corneal thickness, corneal power, and posterior elevation in the long term and im- proves BCVA and anterior elevation. It could improve astigma- tism as well.	none	none
Elbaz et al.	2014	R	Canada	pediatric, adult	off	Accelerated corneal CXL is effective in stabilizing topographic parameters after 12 months of follow-up in mild- moderate keratoconus-affected corneas.	none	none
Hafezi	2021	E	Switzerland, USA, China	N/A	off	Comparison of standard and accelrated protocols, esulted in significantly better VA than the standard protocol.	UNK	UNK
Uysal et al.	2022	R	Turkey	adult	off, on	Similar efficacy of transepithelial DAI-CXL to A-CXL in slowing down the progression of keratoconus in thin corneas without notable effects during a 24-month follow-up period.	no external funding	none
Napolitano et al.	2022	R	Italy, Spain	adult	off	Evaluation of safety and efficacy of customized central corneal regularization (CCR) with simultaneous accelerated corneal collagen cross-linking (A-CXL). The CCR-CXL protocol seems to be safe and effective in arresting corneal ectasia progression and in increasing corneal morphological parameters in keratoconus.	none	none
Kobashi et al.	2018	Rev	Japan	adult	on, off	Although patients in the transepithelial CXL group demonstrated a greater improvement in BSCVA compared with patients in the epi-off CXL group at the 1 year follow-up, transepithelial CXL had less impact on halting progressive corneal ectasia in terms of maximum K than epi-off CXL.	none	none
Bilgihan et al.	2022	R	Turkey	adult	on, off	CXL showed significant improvement in BSCVA and corneal thickness changes, while DAI-CXL maintained corneal thickness. Both groups had reductions in higher-order aberrations, with S-CXL showing more improvement. Spherical aberration only decreased in S-CXL. There were no significant correlations between visual improvements and other metrics.	none	none
Daxer	2008	T	Austria	adult	N/A	The corneal intrastromal implantation surgery technique is easy to perform and appears to be safe and effective for the treatment of moderate and high myopia.	financial interest	financial interest
Kanellopoulos et al.	2019	R	Greece	adult	off	The Athens Protocol demonstrates long-term safety and efficacy for treating corneal ectasia, with stable visual function. Most parameters showed minimal change from 1 year through 10 years after treatment.	none	none

First Author	Year	Study Design	Origin of study	Population	Epithelial removal	Main Findings	Funding Sources	Conflicts of Interest
Grentzelos et al.	2023	R	Greece, UK, India	adult	off	The combination of simultaneous transepithelial phototherapeutic keratectomy (t-PTK) and conventional photorefractive keratectomy (PRK), followed by corneal cross-linking (CXL), appears to be a safe and effective approach for managing progressive keratoconus over a three-year follow-up period.	none	none
Kaiserman et al.	2019	R	Israel	pediatric, adult	off	The Tel-Aviv Protocol is an effective treatment for progressive keratoconus, helping to stop its progression while also enhancing visual acuity and reducing astigmatism.	UNK	none
Hafezi et al.	2021	T	Switzerland, USA, China, Brazil	adult	on	Slit-lamp CXL makes treatment more accessible and affordable by allowing the procedure in clinics without an operating room, reducing costs and expanding availability.	UNK	none
Subasinghe et al.	2018	Rev	New Zealand	pediatric, adult	on, off	When comparing conventional crosslinking with other crosslinking methods, the efficacy of modified techniques still seems to be lower than that with C-CXL. Modified techniques seem to have contradictory results regarding its effectiveness, however, appear to be able to overcome some of the immediate complications of the C-CXL technique.	UNK	none
Sorkin et al.	2014	Rev	Israel	pediatric, adult	on, off	CXL has also been shown to be effective in the treatment of corneal infections, chemical burns, bullous keratopathy and other forms of corneal edema.	none	none
Alhayek et al.	2015	Rev	China	pediatric, adult	on, off	CXL is recommended for patients with early keratoconus who cannot be optically corrected and those who demonstrate recent progression.	Jiangsu Province's Key Provincial Talents Program (RC2011104)	none
Brittingham et al.	2014	R	Switzerland	pediatric, adult	off	The rapid CXL protocol negatively influences the occurrence and depth of the demarcation line 1 month after CXL. Results show a negative effect on the topographical outcome 1 year after CXL.	UNK	UNK
Badawi	2022	R	Egypt	adult	on, off	Significant variations in corneal densitometry in response to the different CXL protocols. The A-CXL group showed a delay in recovering from the haze with persistent increased corneal densitometry. A-CXL appeared to have similar effects, such as the S-CXL protocol, in slowing the progression of KC in adults.TE-CXL showed visual and topographic regression.	no external funding	none
Wajnsztajn et al.	2022	R	Israel	adult	on, off	The non-accelerated epithelium-off protocol was associated with greater flattening of corneal curvature but did not show a better effect on visual acuity as compared to the other CXL protocols.	none	none
Tian et al.	2022	P	China	adult	on	Patients with thinner central corneal thickness and higher Kmax values are more likely to benefit from ATE-CXL.	UNK	none
Aldairi et al.	2022	R	Saudi Arabia	pediatric, adult	off	Both accelrated and standard protocols were effective in stabilizing KC at 9th-month and the last follow-up visit.	none	none
Salman et al.	2021	R	Syria	pediatric, adult	off	A-CXL showed more visual improvement and less pachymetric reduction when compared to the standard protocol. Anterior corneal flattening, posterior corneal steepening, and the change in the posterior astigmatism were significantly higher in the standard protocol; while corneal higher-order aberrations were improved in both protocols.	none	none
Kobashi et al.	2020	MA	Japan	UNK	off	An ACXL shows a comparable efficacy and safety profile at the 1-year follow-up, but it has less impact on improving best spectacle-corrected visual acuity when compared with the Dresden protocol.	none	none
Singh et al.	2020	P	India	pediatric, adult	off	Protocols with hypoosmolar riboflavin and accelerated CXL can reliably be used with adequate outcomes. Safe technique without any significant complications and with good patient acceptability.	none	none
Razmjoo et al.	2017	P	Iran	adult	off	According to topographic criteria and keratometry improvement in the accelerated and conventional protocol are the same. A-CXL is suggested as a safe and effective method.	no external funding	none
Iqbal et al.	2019	RCT	Egypt	pediatric	on, off	SCXL was more effective for paediatric KC and achieved greater stability than either ACXL or TCXL, and ACXL was superior to TCXL. SCXL also achieved marked improvement in both myopia and spherical equivalent and is preferable for management of paediatric KC.	UNK	UNK
Fard et al.	2020	MA	USA	pediatric	on, off	Given its similar efficacy to the conventional protocol and its decreased operative time, the accelerated protocol could be regarded as the preferred technique in pediatric patients. Conversely, transepithelial “epithelium-on” protocol, although safe, is not as efficient.	UNK	UNK
Deshmukh et al.	2019	Rev	India	adult	on, off	Conventional corneal cross-linking (CXL) effectively halts keratoconus progression in corneas 400 microns or thicker, but modifications have been developed for thinner corneas. Hypo-osmolar riboflavin, transepithelial CXL, and customized epithelial debridement offer alternatives for early-stage or thin corneas, while newer techniques like lenticule-assisted CXL and CACXL show promise. Individualized CXL aims to adapt treatment to each patient’s corneal thickness, but the minimum UV dosage required remains uncertain. While most protocols are effective and safe, more extensive studies are needed to confirm long-term outcomes.	none	none
Vinciguerra et al.	2021	Rev	Italy	adult	off	Transepithelial CXL reduces pain and complications while aiming for S-CXL effectiveness. I-CXL has improved with refinements in riboflavin, irradiation, and oxygen use. The latest protocol shows results closer to S-CXL, making it a safer option for early keratoconus, though further studies are needed.	UNK	none
Liao et al.	2019	R	China	pediatric, adult	off	This study compared 5-minute and 10-minute iontophoresis-assisted CXL (I-CXL) for keratoconus in 42 patients. After 12 months, the 10-minute group showed better visual acuity improvement, a greater reduction in Kmax values, and a deeper demarcation line on OCT. I-CXL for 10 minutes was more effective in halting keratoconus progression, but further long-term studies are needed to assess its safety and efficacy.	Natural Science Foundation of Hunan Province (No.2016JJ2163); Natural Science Foundation of Hubei Province (No.2015CFC837); Health and Family Planning Committee Science Foundation of Hubei Province (No.WJ2015MB259); Health and Family Planning Committee Science Foundation of Wuhan Municipality (No.WX17A13).	none
Chan et al.	2013	Rev	Australia	adult	off	Corneal cross-linking can alter the course of keratoconus and potentially halt its progression. Early detection and monitoring are crucial to ensure patients receive timely treatment. Future research may optimize the procedure, improving accessibility and reducing the need for more invasive interventions.	UNK	UNK
Bibkova et al.	2014	P	Russia	adult	off	Iontophoresis-assisted transepithelial CXL may improve riboflavin absorption, shorten procedure time, and enhance patient comfort. However, further long-term studies are needed to assess its effectiveness and safety.	UNK	none
Cantemir et al.	2017	R	Romania	adult	on, off	I-CXL is as effective as epi-off CXL in halting early keratoconus progression while offering greater safety and faster visual recovery.	UNK	UNK
Vinciguerra et al.	2022	R	Italy	adult	off	Long-term outcomes of I-CXL show a 26% progression rate with limited improvement in morphology and function. This suggests that I-CXL may be more suitable for less aggressive cases or for patients with slower progression, and newer iontophoresis protocols with higher energy may be considered.	NONE	R.V. and P.V. are consultants for OCULUS Optikgeräte GmbH, P.V. is a consultant for Schwind, R.V. is a consultant for OPTIMO MEDICAL.
Bilgihan et al.	2017	CoS	Turkey	adult	on, off	The DAI-CXL protocol appears to be as effective as the C-CXL protocol in stopping keratoconus progression after one year of follow-up	none	UNK
Ziaei et al.	2019	P	New Zealand	pediatric, adult	off, on	Clinical studies have reported short-term favourable outcomes with both accelerated and transepithelial crosslinking protocols.	UNK	none
Mazzotta et al.	2014	CoS	Italy	pediatric, adult	off	In vivo confocal microscopy and corneal OCT revealed detailed corneal changes after epithelium-off PL-ACXL and CL-ACXL treatments. Pulsed light treatments showed a higher apoptotic effect in the corneal stroma. Long-term follow-up and larger studies are needed to determine the clinical efficacy of ACXL.	UNK	none

First Author	Year	Study Design	Origin of study	Population	Epithelial removal	Main Findings	Funding Sources	Conflicts of Interest
Mazzotta et al.	2014	P	Italy	pediatric, adult	off	Pulsed and continuous light accelerated cross-linking are safe, evolving methods for stabilizing keratoconus in a shorter treatment time. However, their efficacy requires further investigation with mid- to long-term follow-up and larger patient cohorts.	UNK	none
Jiang et al.	2017	CoS	China	adult	off, on	Both CXL and pl-ACXL are safe and effective for stabilizing keratoconus progression. While CXL provides better visual and topographic outcomes, pl-ACXL causes less microstructural damage. Long-term effects of both methods need further study.	UNK	UNK
Yousif et al.	2023	R	egypt	UNK	off	The study concluded that longer-duration cl-CXL is as effective as pl-CXL in terms of visual, refractive, and keratometric outcomes, as well as corneal tissue penetration, in a large group of A-CXL-treated eyes	none	none
Abdel-Radi et al.	2023	P	egypt	adult	off	Accelerated pulsed high-fluence CXL caused minimal corneal endothelial changes, with stable endothelial cell count and no significant morphological alterations.	none	none
Hernandez-Camarena et al.	2019	R	mexico city	pediatric, adult	off	This study found that both pulsed A-CXL protocols effectively stabilized or improved topography in over 85% of cases after 12 months, with no correlation between demarcation line depth and outcomes. However, further long-term studies with larger samples are needed.	UNK	none
Hafezi et al.	2022	R	-	pediatric, adult	off	This study found that the demarcation line depth in accelerated epi-off CXL is independent of the patient's position. Further research comparing demarcation line depths with UV irradiation in different positions using the same riboflavin solution is needed to confirm these findings.	Light for Sight Foundation, Zurich, Switzerland and the Velux Foundation, Zurich, Switzerland. NL received funding as a PhD student from the Chinese Scholarship Council, Beijing, China.	FH holds a patent on a UV light source (PCT/CH 2012/000090). NH is CEO of EMAGine AG, a company producing a CXL device.
Peyman et al.	2021	LR	Iran	N/A	off	Concern about infectious corneal ulcers after CXL, possibly attributable to the intraoperative contamination.	UNK	UNK
Hafezi et al.	2021	LR	Switzerland	N/A	off	The authors do not believe that CXL performed in an office or oper- ating room–based setting increases the risk of corneal infection.	Light for Sight Foundation and Velux Stiftung, Zurich, Switzerland	F. Hafezi has patent on a UV light source (PCT/CH 2012/000090). N. Hafezi is CEO of EMAGine AG, a company producing a CXL device.
Daxer et al.	2010	P	Austria	adult	N/A	ICCR implantation significantly improved visual function in keratoconus patients, with both UDVA and CDVA improving during the first postoperative year. The nomogram for treatment requires grading the disease using only K readings.	none	UNK
Bibkova et al.	2018	R	Russia	adult	N/A	Both MyoRing implantation and MyoRing combined with CXL were effective for treating keratoconus. After 36 months, the MyoRing + CXL group showed slightly better outcomes, while the MyoRing-only group maintained stable visual and refractive results.	UNK	none
Krueger et al.	2010	CR	USA	adult	off, on	Partial topography-guided PRK with CXL safely stabilizes keratoconus, improves vision, and reduces aberrations, though results may be limited in very steep or thin corneas.	none	none
Kymionis et al.	2012	CoS	Greece	adult	off, on	Epithelial removal through t-PTK during CXL leads to improved visual and refractive outcomes compared to mechanical epithelial debridement.	none	UNK
Grentzelos et al.	2019	CoS	Switzerland, Greece	adult	off, on	The combined approach of transepithelial PTK and conventional PRK, followed by simultaneous CXL, effectively stabilized the cornea and improved vision in keratoconic patients.	none	UNK
Grentzelos et al.	2017	P	Switzerland, Greece	adult	off, on	The combination of transepithelial PTK, conventional PRK, and CXL effectively stabilized the cornea and improved vision in keratoconus patients.	none	UNK
Rabina et al.	2020	R	Israel	adult	off	The Tel-Aviv Protocol effectively improved visual acuity and astigmatism while halting keratoconus progression in progressive keratoconus patients.	UNK	UNK

BCVA= best corrected visual acuity, BSCVA = best spectacle-corrected visual acuity, CDVA = corrected distance visual acuity, CoS = comparative study, CR = case report, CS = clinical study, CT = clinical trial, D = diopters, E = editorial, IOP = intraocular pressure, LR = letter reply, MA = meta-analysis, P = prospective, RCT = randomized controlled trial, Rev = review, R = retrospective study, T = technique, UDVA = uncorrected distance visual acuity, VA = visual acuity
